# Underexplored maternal microbiomes: immune, metabolic, and microbial pathways shaping pregnancy outcomes

**DOI:** 10.1128/iai.00608-25

**Published:** 2026-02-09

**Authors:** Rafael Tomoya Michita, Nicole Jimenez, Melissa M. Herbst-Kralovetz, Indira U. Mysorekar

**Affiliations:** 1 Department of Medicine, Section of Infectious Diseases, Baylor College of Medicine 171841, Houston, Texas, USA; 2 Department of Obstetrics and Gynecology, College of Medicine-Phoenix, University of Arizona 42283, Phoenix, Arizona, USA; 3 Department of Basic Medical Sciences, College of Medicine-Phoenix, University of Arizona 42283, Phoenix, Arizona, USA; 4 Department of Molecular Virology and Microbiology, Baylor College of Medicine 3989, Houston, Texas, USA; 5 Huffington Center of Aging, Baylor College of Medicine 3989, Houston, Texas, USA; University of California Davis, Davis, California, USA

**Keywords:** gut microbiota, oral microbiome, respiratory microbiome, urinary microbiota, cervicovaginal microbiota, maternal microbiome

## Abstract

Maternal microbial ecosystems play critical roles in shaping reproductive physiology and pregnancy outcomes. During the pre-conception and prenatal periods, these communities modulate maternal physiology by regulating immune tolerance, nutrient metabolism, and susceptibility to pregnancy complications such as preterm birth, hypertensive disorders, and gestational diabetes. While the gut microbiota has been extensively studied, the roles of cervicovaginal, urinary, respiratory, oral, and upper reproductive tract microbiomes remain less clear. In this minireview, we synthesize current knowledge on these underexplored maternal microbiomes, with an emphasis on the cervicovaginal and urinary microbiota and their interactions with the placenta and fetus. We discuss cross-niche microbial signaling, the role of environmental and social determinants in shaping these ecosystems, and mechanisms by which microbes or their products influence host physiology without direct colonization. We also consider the translational potential of microbiota-based interventions to safely improve pregnancy outcomes. Finally, we identify major knowledge gaps and research priorities necessary to advance a more integrated understanding of maternal microbial influences on reproductive and neonatal health. Our synthesis reframes the maternal microbiome as a coordinated, multi-site network that modulates systemic immune and metabolic pathways critical for reproductive success. Understanding these connections will open new avenues for predicting, preventing, and treating pregnancy-related disorders through precision microbiome science.

## INTRODUCTION

The human microbiome consists of diverse microbial communities that inhabit specific body sites, determined by unique physio-chemical properties and host-microbe interactions. These communities differ from one person to another and can change over time (1), thus influencing the host’s metabolome and immune system (2, 3). Although the definition of a healthy microbiome remains elusive, it is increasingly recognized that intra- and inter-individual microbial diversity is also influenced by the major histocompatibility complex (MHC) (4, 5). Throughout pregnancy, the maternal microbiota plays a vital role in fostering a symbiotic relationship that is essential for the health of both the mother and the fetus. From conception to birth, several variables, including physical, physiological, environmental, social determinants of health, and hormonal alterations, exert selective pressures on the maternal microbiota (6–8) to meet the physiological needs of the developing fetus.

The maternal microbiome, particularly the cervicovaginal and gut microbiota, produces bioactive compounds that support host immune regulation, metabolic functions, and protection against pathogens (7, 9–11). Primarily influenced by local environmental conditions (e.g., the body site and hormonal changes), the full extent of the maternal microbiome changes and their effect on pregnancy remains unclear. Given the role of the gut microbiota in host metabolism, gut microbial diversity contributes to a broader range of metabolites (such as short-chain fatty acids [SCFAs], secondary bile acids, and dietary precursors) that influence placental and fetal development (9, 12–14). In contrast, the cervicovaginal microbiota is less diverse primarily due to the acidic pH maintained by lactic acid-producing *Lactobacillus* spp., which, along with hormonal and immune-stimulating factors (15), protect against urogenital diseases (16). While research has largely focused on the gut and vaginal microbiota, our understanding of microbiome dynamics in other body sites (cervicovaginal, urinary, respiratory, oral, upper reproductive tract remain less clear) during pregnancy remains limited. Similarly, the influence of the parental microbiome prior to conception is not well understood, but emerging evidence underscores its importance for pregnancy health and is likely to gain increasing attention in future research.

Pathological changes in the maternal microbiome composition, known as dysbiosis, have been associated with several adverse pregnancy outcomes, including preterm birth (PTB), miscarriage, preeclampsia, and gestational diabetes mellitus (GDM) (17–21). As more studies investigate the complex interactions between the maternal microbiome and pregnancy outcomes, it is worth noting the ongoing debate over the existence of the fetal microbiota (22–27). Despite this lack of consensus, it is now widely recognized that microbiota-derived components, rather than live bacteria, are transferred from the mother to the fetus (e.g., the maternal-fetal gut microbiota axis), influencing fetal development and priming postnatal colonization (14, 28–31).

**Fig 1:**
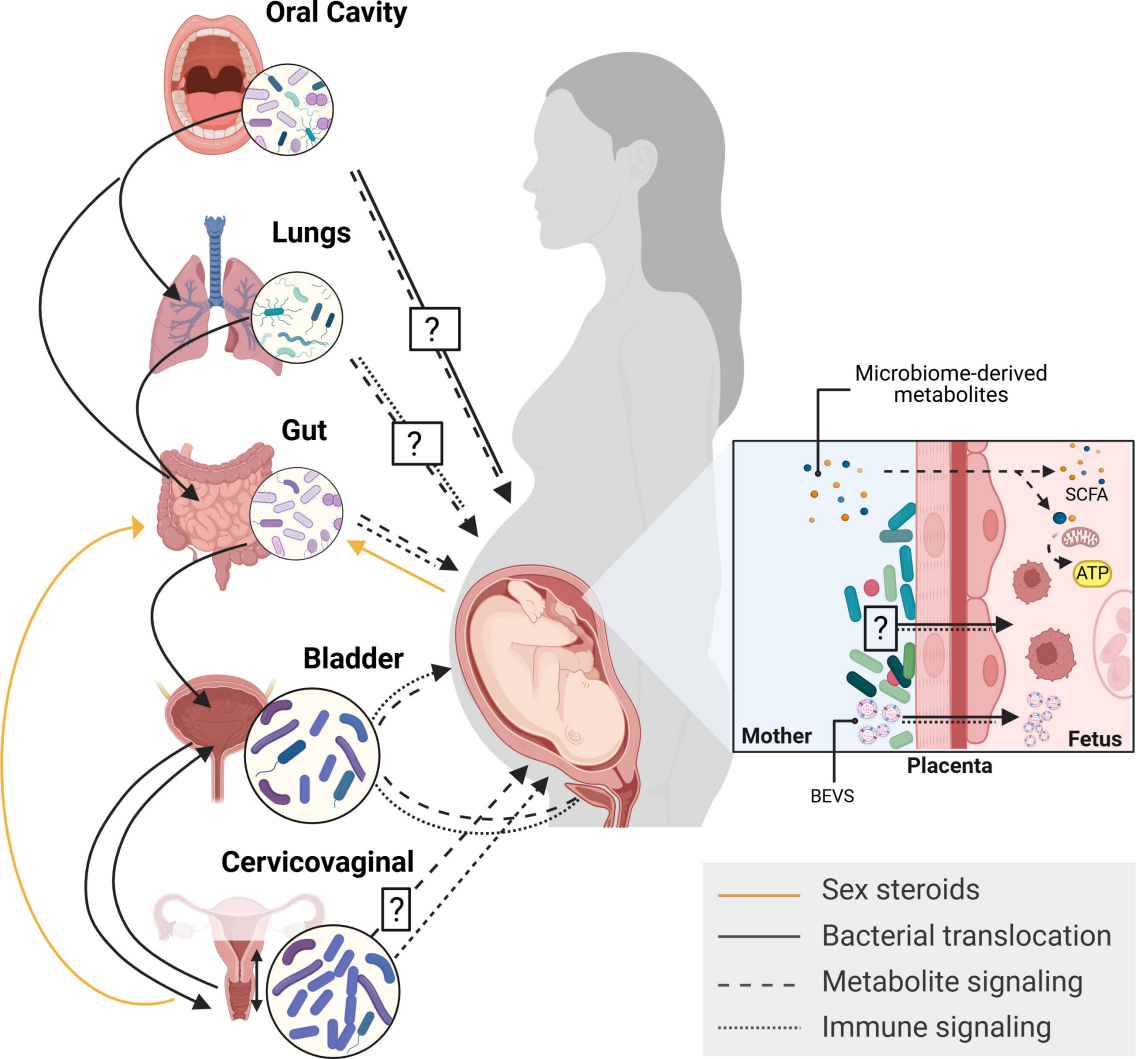
Pregnancy-associated shifts in maternal microbiota across multiple body sites can signal to the placenta, influencing its function and fetal development. The maternal microbiome spans multiple body niches, including the oral cavity, lungs, gut, bladder, cervicovaginal tract, and placental basal plate, whose composition is shaped by hormonal, immune, anatomic, and metabolic changes throughout gestation. The maternal skin also represents an underexplored niche. Microbes and their metabolites influence distal compartments through bacterial translocation, immune and metabolic signaling, and endocrine feedback. During pregnancy, gut microbes metabolize circulating pregnancy hormones such as estrogen (via the estrobolome) and progesterone, modulating systemic endocrine balance and microbial composition across body sites. Microbiome-derived metabolites, such as short-chain fatty acids (SCFAs) and bacterial extracellular vesicles (BEVs), may act on distant maternal body sites and cross the placenta, shaping fetal development and immune priming. Bidirectional interactions between sex steroids and microbial communities, together with microbial trafficking between sites, highlight the dynamic of the maternal microbiome and the need to define microbial reservoirs influencing pregnancy outcomes. While metabolite and immune signaling from the gut to the placenta is well supported, the impact of the oral, pulmonary, cervicovaginal, and placental basal plate microbiomes on placental and fetal development remains to be mechanistically elucidated. Shared microbiota between oral cavity, lungs, and the gut supports the concept of bacterial swapping among niches, whereas translocation from the gut or rectum to the cervicovaginal tract can promote dysbiosis. Similarly, uropathogenic *E. coli* may traffic between the gut, vagina, and bladder, establishing reservoirs for bacterial vaginosis and recurrent urinary tract infections.

This review provides an overview of the maternal microbiome and its impact on pregnancy, from the pre-conception period to the perinatal stage. While not exhaustive, we highlight key findings and emerging insights across distinct microbiome niches (Fig. 1). We begin with the bidirectional relationship between female hormones and microbial communities and their combined influence on reproductive health. Although we touch on the topic of gut microbiota, which has been extensively reviewed elsewhere (28), our focus is on less explored body niches, particularly the cervicovaginal and urinary microbiomes, their roles in disease states, and how they are shaped by obstetric history, hormonal changes, sexually transmitted infections (STIs), and social determinants of health. We further examine microbial interactions at the maternal-placental interface and their potential influence on fetal development.

## MICROBIOME AND HORMONAL ADAPTATIONS DURING PREGNANCY

Physiological and anatomical changes during pregnancy affect several organ systems, including the reproductive, respiratory, endocrine, gastrointestinal, circulatory, hematologic, and renal systems (32). These adaptations significantly influence the composition of the maternal oral, cervicovaginal, and gut microbiomes (32, 33). Pregnancy hormones (e.g., human chorionic gonadotropin, human placental lactogen, estrogen, and progesterone) also affect the maternal microbiota or vice versa, highlighting a complex bidirectional relationship (34, 35). It is also known that the microbes in the gut regulate circulating estrogen levels by the estrobolome, a collection of genes encoding enzymes that deconjugates estrogen from glucuronic acid, affecting female reproductive health (36). During gestation, the “progesterone block effect” suppresses myometrial contractility by downregulating contraction-associated proteins, such as oxytocin receptors, connexin-43, and prostaglandin receptors (37, 38). This effect preserves uterine quiescence, helping to prevent premature contractions and miscarriage while providing a stable environment for fetal growth. In addition to its uterine effects, progesterone-induced smooth muscle relaxation impacts the gastrointestinal tract by reducing motility, the vascular and respiratory systems through vasodilation and bronchodilation, and the urinary tract by decreasing bladder tone. Collectively, these changes influence the growth and composition of microbial communities across these body sites. However, the mechanistic understanding of how individual or combined hormonal changes impact microbiota composition over time, and the contribution of microbial-derived metabolites to pregnancy maintenance, remains limited. Of note, microbial endocrinology has emerged as a field dedicated to understanding bidirectional communication between the microbiota and the host endocrine system (34).

In this context, studies investigating the effects of pregnancy hormones on microbial dynamics within reproductive compartments have begun to uncover potential mechanisms. The follicular fluid from women undergoing *in vitro* fertilization (IVF) has been shown to harbor biofilm-forming bacteria (39). Given these hormone-microbe interactions, IVF treatment cycles traditionally involve controlled ovarian stimulation with exogenous recombinant hormones to stimulate follicle maturation, promote oocyte maturation and ovulation, and support the luteal phase to optimize embryo implantation. It is noteworthy that supraphysiological IVF hormonal can systemically affect the microbiome, with studies reporting shifts in the vaginal, endometrial, and gut microbiota, although the clinical relevance and timing of these microbial changes remain unclear (40–42).

Additionally, evidence shows that microbial gut-derived metabolites induce secretion of gut hormones that act on the hypothalamic-pituitary-gonadal (HPG) axis for FSH and LH secretion, both involved in follicle maturation (43, 44). Consistent with this, estradiol and progesterone modulate the growth of specific bacterial taxa in the fluid of ovarian follicles, including *Lactobacillus* spp., *Streptococcus* spp., and *Bifidobacterium* spp., while inhibiting the growth of *Escherichia coli* and *Streptococcus agalactiae* (39).

At the phylum level, the upper female reproductive tract (endometrium and fallopian tubes) is dominated by Proteobacteria, Actinobacteria, and Bacteroidetes, in contrast to the Firmicutes-rich lower tract (45). This gradient in microbial composition suggests distinct yet poorly understood functions at the community level along the reproductive tract. Although some taxa may support reproductive function, a recent meta-analysis found that bacterial colonization of follicular fluid in infertile women undergoing IVF was associated with lower clinical pregnancy rates. However, the evidence is limited, and no specific taxa were consistently linked to outcomes due to study heterogeneity (46). Conflicting results could reflect the cervicovaginal health, as *Lactobacillus* spp. predominance is associated with IVF success (47, 48). Notably, the diversity of follicular fluid microbiota positively correlates with the diversity at the MHC *loci* (5), mirroring findings in the gut microbiota (4), a biological variable often overlooked in reproductive microbiome studies.

Thus far, the role of microbiota in the ovaries and other reproductive niches is poorly understood, and it remains unclear whether colonization of these niches is beneficial, harmful, or incidental (49). However, emerging findings in germ-free mouse models demonstrate that gut microbial colonization and associated SCFAs (acetate, butyrate, and propionate) can influence ovarian reserve, pointing to a previously underappreciated gut-ovary communication axis (50).

## MICROBIOME DYNAMICS OF THE RESPIRATORY TRACT DURING PREGNANCY

Studies of the respiratory tract microbiome during pregnancy are limited. In mice, determining pregnancy-related effects on lung microbiota is complicated by housing conditions and species-specific behaviors such as coprophagy (51). And in humans, studies of the oral upper respiratory tract and the upper female reproductive tract have been inhibited by contamination and inherent low-biomass (52, 53). Despite these limitations, pregnancy has been found to increase microbial diversity in the upper respiratory tract of first-trimester pregnant women (54). While overall upper respiratory microbiome composition of pregnant and non-pregnant women is similar (Actinobacteria, Proteobacteria, and Firmicutes), parity associates with distinct taxa, including higher *Moraxella* abundance in multiparous women and increased *Corynebacterium* in primiparous women (54). Also, maternal cohabitation with offspring has been associated with maternal microbial convergence (mothers acquiring child-associated bacteria) in the upper respiratory tract (54). Viral infections during pregnancy, such as SARS-CoV-2, have also been associated with increased diversity of the nasopharyngeal microbiome and members of *Bacteroidetes* and *Tenericutes* (55). Notably, these microbial shifts were suggested to persist in women with past SARS-CoV-2 infection (55).

It is proposed that the human lung microbiome is closely interconnected with the oral and gut microbiota, forming a cross-compartmental network that influences respiratory and systemic health (56, 57). While there is a limited understanding of the mechanisms by which pregnancy modulates the cross-compartmental network of microbiomes, a recent systematic review suggests the view that the oral microbiota is largely stable throughout pregnancy (58). However, significant differences in abundance of core bacteria, such as *Streptococcus*, *Porphyromonas*, and *Haemophilus*, have been reported (59). The association between the oral microbiota during pregnancy and maternal systemic health and birth outcomes remains inconclusive (58) and warrants further investigation.

## MICROBIOTA-DRIVEN MODULATION OF PREGNANCY AND FETAL HEALTH

In mouse studies, progesterone has been shown to modulate gut microbiota composition, leading to an increased abundance of SCFA-producing *Bifidobacterium* species in pregnant females (60). In humans, the gut microbiota of pregnant women is enriched in members of the phylum Firmicutes (e.g., butyrate producers) during the first trimester, while the third trimester shows increased Proteobacteria (often associated with inflammation) and Actinobacteria (often involved in carbohydrate fermentation and SCFA production) (61). Remarkably, germ-free mice gut-colonized with third-trimester microbiota from pregnant women exhibited increased adiposity and inflammation, potentially supporting fetal growth (61). One study in germ-free pregnant mice has provided novel insights into how *Bifidobacterium* species regulate placental metabolism and endocrine function (62). Another reported that vancomycin-induced gut dysbiosis, characterized by increased *Bacteroidetes* and decreased *Firmicutes*, impairs placental NK cell effector functions compared to controls (63).

Alterations in gut microbiota composition and associated microbial metabolites have been linked to immune modulation during pregnancy (28) and shown to support healthy placentation in mouse models (14, 63). The intestinal symbiont *Akkermansia muciniphila* (64) secretes bacterial extracellular vesicles that promote placental development and mitigate preeclampsia-like phenotypes in a mouse model (65). This bacterium also shifts the gut microbiome toward more metabolically active, SCFA-producing Firmicutes during pregnancy (66). In *A. muciniphila*-exposed dams, elevated SCFA and amino acid serum levels were found to affect brain development and the differentiation of intestinal stem cells (66). This study is consistent with the role of the maternal microbiome in mouse fetal brain development (67). Additionally, gut microbes are known to produce several stem cell differentiation factors, including SCFAs; organic acids such as lactate and succinate; indole derivatives; and secondary bile acids (68).

Maternal gut microbiota, specifically *Bifidobacterium* spp. and their metabolite inosine, can epigenetically program offspring T-cell antiviral immunity (69, 70). Additionally, *Eggerthella lenta* has been found to metabolize bile acid glucocorticoids that act on gamma-aminobutyric acid (GABA) receptors (71), which, when reduced, are associated with post-partum depression (72). Another gut commensal, *Bacteroides fragilis*, has also been found to inhibit the bile acid sensor, FXR (farnesoid X receptor), contributing to intrahepatic cholestasis of pregnancy (73). Of note, several bile acid derivatives are found to be unique to pregnancy (74). However, how these metabolites are processed by microbiota and their resulting biological roles remains poorly understood. Beyond gut-derived metabolites, bacterial-derived extracellular vesicles (75) are increasingly recognized for their ability to cross the placenta and reach the intra-amniotic space (76, 77).

The microbiome not only affects pregnant women and the developing fetus but also plays a role in preconception. In mice, paternal gut microbiome disruptions caused by antibiotics or osmotic laxatives impair germline cells and lead to adverse fetal outcomes by impacting both sperm biology and placental function (78). Strikingly, these intergenerational effects are reversible if the paternal gut microbiota is restored before conception (78). These results highlight a previously overlooked contribution of the paternal microbiome to pregnancy, fetal development, and reproductive health. Although not fully understood, the link between gut bacteria (but not limited to) and reproductive function suggests that microbiome disruptions in both parents may contribute to adverse outcomes in the offspring. This expands the relevance of microbiome research within the scope of the Developmental Origins of Health and Disease (DOHaD) research field (79, 80).

## CERVICOVAGINAL MICROBIOME IN PREGNANCY AND HEALTH

Vaginal health is characterized by low bacterial diversity dominated by a few *Lactobacillus* spp. (81), in contrast to the gut microbiome, where high diversity is associated with intestinal health. When the cervicovaginal microbiome is depleted of these lactobacilli, and anaerobic bacteria are increased, this is deemed dysbiosis and is linked to the most common gynecologic condition: bacterial vaginosis (BV) (81). This paradigm of health is observed across disease states and conditions, including PTB (82).

**Fig 2:**
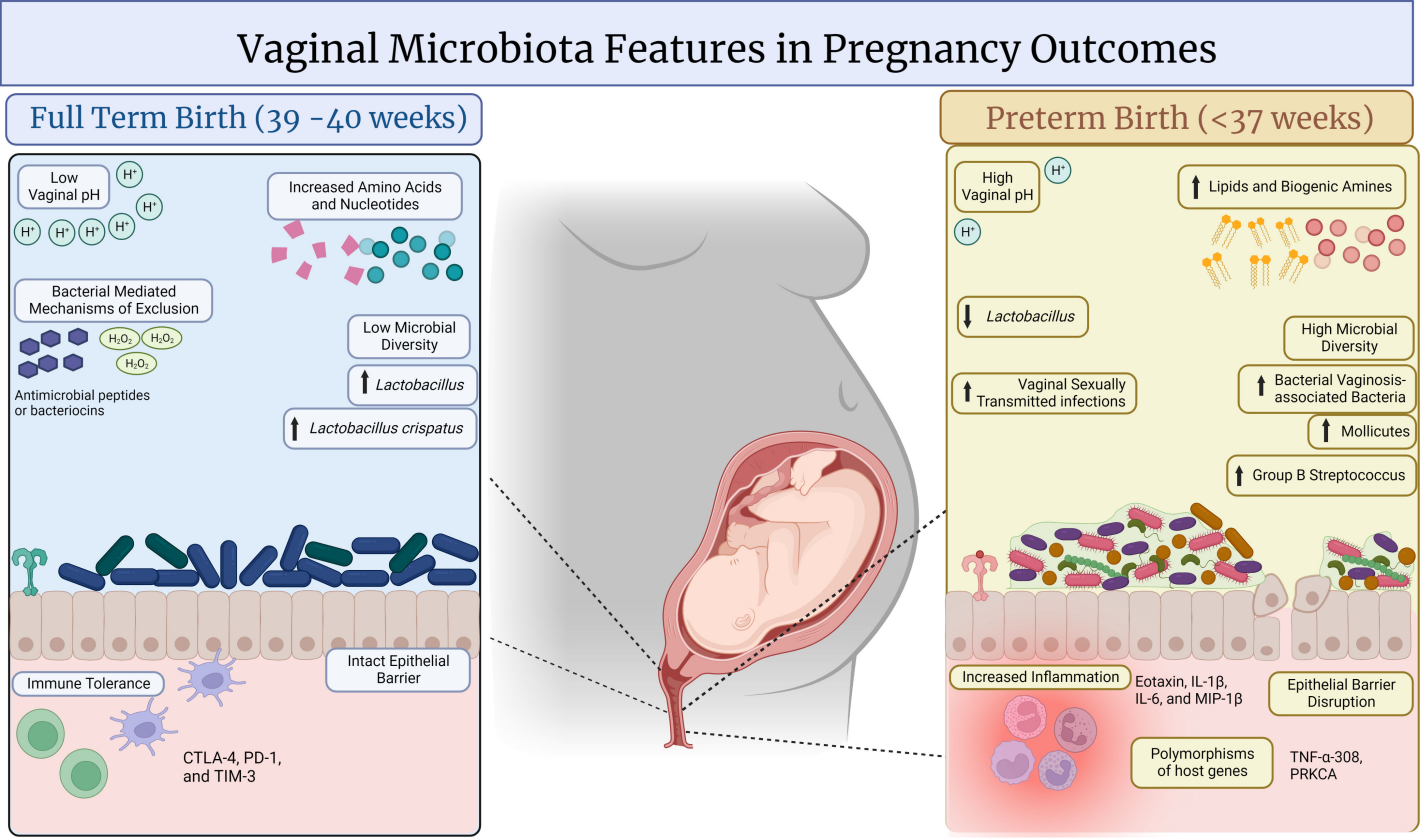
Cervicovaginal microbiota and maternal factors associated with pregnancy outcomes. The cervicovaginal microenvironment in humans, with its amino acids and carbohydrate metabolites, is linked with term birth and preterm birth (PTB). Women with term deliveries often have a cervicovaginal microenvironment rich in healthy *Lactobacillus* species and low microbial diversity. These metabolites, from lactobacilli, promote cervicovaginal homeostasis by producing lactic acid, which acidifies the local microenvironment. *L. crispatus* is associated with favorable pregnancy outcomes and health. Additional exclusionary byproducts from *Lactobacillus* species can stimulate the host to produce hydrogen peroxide, antimicrobial peptides, and anti-inflammatory cytokines and improve barrier integrity. In contrast, PTB is associated with a decrease in health-associated lactobacilli and increased microbial diversity and vaginal pH. Dysbiotic vaginal bacteria, such as bacterial vaginosis-associated bacteria (BVAB) (*Atopobium*, *Gardnerella*, *Gemella*, *Prevotella*, *Megasphaera*, BVAB1, and *Sneathia*), pathobionts (Ureaplasma, Mycoplasma, and Group B Streptococcus), and known sexually transmitted infection pathogens (HIV, HPV, *Trichomonas vaginalis*, and *Chlamydia trachomatis*), are also frequently observed in vaginal microbiome profiles of PTB. Also, increased lipids and biogenic amines related to BVAB are found in PTB. These microorganisms trigger inflammation, and putative microbial products, such as sialidase, affect epithelial barrier function. Further, host genetic factors that also may be important in host-microbe interactions, such as genetic variant in the tumor necrosis factor-alpha (TNF-α), a proinflammatory cytokine, have also been implicated in increased risk for PTB.

The abundance and stability of lactobacilli during pregnancy are thought to be driven by estrogen levels and have been associated with better pregnancy health outcomes (83). In contrast, progestins have been associated with suppression of vaginal lactobacilli, presumably by altering cell-free glycogen levels in the vaginal fluid, which are broken down by host and bacterial glycoside hydrolases into maltodextrins used by lactobacilli under anaerobic conditions (84–86). Throughout pregnancy, the cervicovaginal microbiome shifts in response to physiological and hormonal changes. Several human studies have found that pregnant women exhibit a higher prevalence and abundance of lactobacilli in the cervicovaginal microbiome compared to nonpregnant women (87) (Fig. 2). Additionally, the late trimester has been associated with increased lactobacilli dominance compared to earlier trimesters (88). Thus, it has been proposed that lactobacilli contribute to successful birth outcomes and protect the cervicovaginal environment from infection during pregnancy (89, 90). Lactobacilli may be integral to cervicovaginal homeostasis not only through production of antimicrobial compounds (91, 92), but also by interacting with the host and strengthening mucosal barrier integrity (93, 94), promoting host innate immunity and dampening host inflammation through its peptidoglycan and signaling peptides (16, 92). The hormonal impact on the cervicovaginal environment is even observed at the molecular level, with metabolic changes starting as early as the first trimester (11). As gestation progresses, levels of lactate and amino acids increase, while glucose, organic acids, and biogenic amines decline (11) (Fig. 2). Whether or not these signatures derive from the host alone, the microbiome, or both is being investigated further. In addition, it is thought that the vaginal microbes may be important for promoting the infant’s immune system (95). Due to these linkages, many human studies have investigated the transmission of microbes from mother to infant and the potential seeding of the neonate and infant gut microbiome (81, 96), but studies are still ongoing to determine the long-term impact of this on the infant (97). One factor that may influence successful pregnancy and neonate outcomes is increased parity or mothers who have previously given birth (98). Multiparity is associated with loss of vaginal lactobacilli, whereas longer gestation (37–42 weeks) correlates with higher abundance, particularly in nulliparous women (98).

The cervicovaginal microbiome is suggested as a key player in the risk of PTB and other gynecologic sequelae (98), as it undergoes significant changes postpartum, becoming more diverse and depleted in lactobacilli (81, 99, 100). However, the microbiome can revert to its previous lactobacilli-dominant state with time (81, 99). This change in composition is primarily mediated by pregnancy hormones, such as estrogen, that drive tissue structure remodeling and glycogen deposition (101). Estrogen also plays a key role in maintaining *Lactobacillus* spp. in both the vaginal and urinary microbiota (86).

The cervicovaginal environment can also be altered due to other factors beyond pregnancy (87), including STIs (102), antibiotic usage (103), cigarette smoking (104), hormonal therapy (101), environmental exposures (105), and menopausal status (106). Some of these factors can directly affect microbes; for instance, antibiotic usage and cigarette smoking can release lysogenic bacteriophages and deplete health-promoting lactobacilli (107). Other factors impact the host microenvironment, including hormonal changes, either prescribed or naturally occurring throughout a woman’s life span, which can increase or decrease glycogen deposition (106). Reproductive history also exerts lasting effects on maternal microbiota composition, possibly mediated by labor-associated inflammation, hormonal shifts, and immunological memory in the vaginal environment (98), although other sites may be involved. Nevertheless, the cervicovaginal environment can revert to its original microbial composition with minor changes to the microenvironment (108), or it can shift to a more diverse state characterized by a decrease in lactobacilli, making it more similar to BV.

## RISKS AND ADVERSE PREGNANCY OUTCOMES RELATED TO THE VAGINAL MICROBIOME

Adverse pregnancy outcome risk factors include a history of STIs (109) and BV (110), maternal parity (111, 112), individual or family history of PTB (113), environmental factors (tobacco/drug use, phthalates exposure) (114), and psychosocial or perceived stress (115). Of note, prenatal stress causes vaginal dysbiosis that may seed neonatal microbial communities, shaping early immune and developmental outcomes (116). Perturbations in the cervicovaginal microbiome have been associated with PTB risk through mechanisms involving hormone dysregulation (117), inflammation (118), and infections (102). Several pathogenic microorganisms have been associated with an increased risk for PTB, including Group B *Streptococcus*, *Trichomonas vaginalis*, *Chlamydia trachomatis*, *Neisseria gonorrhoeae*, *Ureaplasma*, *Mycoplasma*, human papillomavirus (HPV), and human immunodeficiency virus (HIV) (119). However, through next-generation sequencing studies, we now know that many more bacteria and communities of bacteria can contribute to adverse pregnancy outcomes. Additionally, communities of associated organisms and conditions, including BV, drive dysbiosis and shift the microbiome toward a disease state, which may increase the risk for these adverse pregnancy outcomes (120) (Fig. 2). Interestingly, growing evidence suggests that polymicrobial conditions can be exchanged between partners, highlighting additional factors to consider in pregnancy complications and treatment strategies (121, 122).

Studies have observed depletion in *Lactobacillus crispatus* and *Lactobacillus jensenii* and an increase of BV-associated bacteria (BVAB), including *Sneathia*, *Prevotella*, *Gardnerella*, *Ureaplasma*, and *Mycoplasma*, which are associated with PTB and spontaneous rupture of membranes (Fig. 2) (17, 123–125). There has been growing appreciation for the role of the cervicovaginal microbiome in host interactions, particularly its impact on immune and metabolic regulation (17, 105). Metabolites associated with PTB, such as depletion in lactate, succinate, and carbohydrates, as well as enrichment of polyamines, fatty acids, and xenobiotics (126–128) (Fig. 2), are associated with decreased vaginal lactobacilli and increased abundance of BVAB. In addition, immunoproteomic signatures of PTB have indicated an increase in proinflammatory cytokines (129) and metabolites associated with inflammation (130) in the cervicovaginal environment (131) (Fig. 2). It is well established that dysbiotic cervicovaginal microbiota alters the immunometabolic microenvironment (132–134), in part through bacterial extracellular vesicles enriched in immune-stimulatory proteins (135, 136) that trigger responses across the lower and upper reproductive tract (137). Additionally, some BVAB have putative capability to ascend from the vagina to the uterus, causing intrauterine infection that leads to inflammation and prostaglandin overproduction, which in turn triggers contractions (138). In addition to bacteria, Torque teno viruses are associated with increased inflammation during pregnancy, which may also contribute to PTB risk (139). This has also been observed with yeast species (140). Further studies are needed to understand interkingdom interactions between virome, mycobiome, and protozoan communities with bacteria, which may influence pregnancy outcomes. For example, recent work has identified fungal-bacterial-host interactions in the endometriosis progression in humans, with findings validated in mouse models (141).

Beyond microbial and host interactions, sociodemographic factors also shape reproductive outcomes. It has been suggested that the cervicovaginal microbiome may contribute to the health disparities observed in PTB (142, 143). However, sociocultural, economic, political, educational, psychosocial stress, or historical structural drivers of these populations may also be important risk factors to investigate further (144). Psychosocial stress, in particular, has been associated with increased PTB risk (145–147) and may influence the cervicovaginal microbiome through stress-related inflammatory pathways (148), potentially promoting BV (149). Integrating social determinants of health with maternal microbiome studies will be essential to better understand and address the global disparities in reproductive health outcomes.

## MODULATION OF THE VAGINAL MICROBIOME FOR BETTER PREGNANCY OUTCOMES

**Fig 3:**
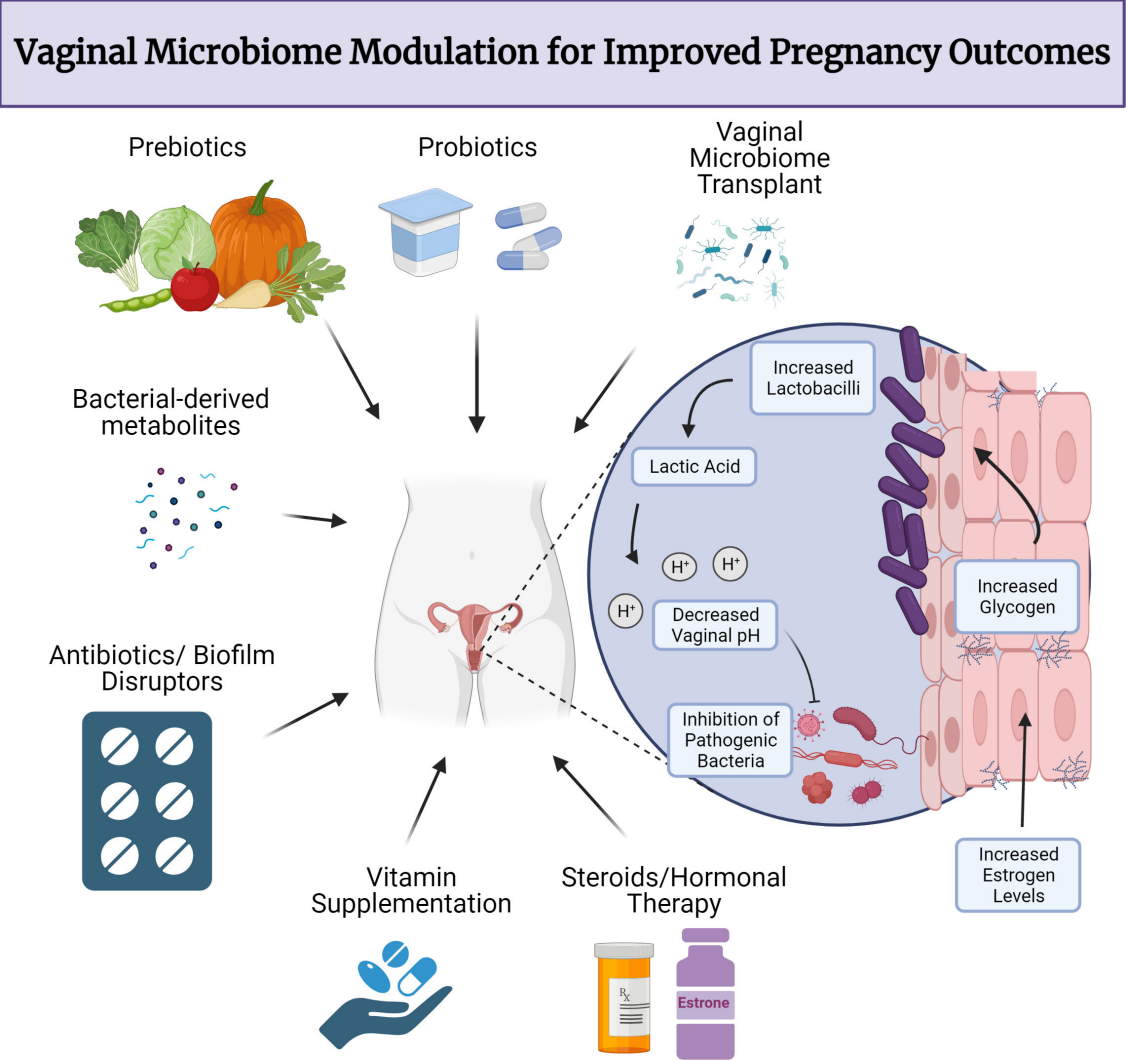
Modulating the human vaginal microbiome for better pregnancy outcomes. Current and proposed therapies for the alteration of microbial composition to increase reproductive success. The vaginal microbiome can serve as a therapeutic target for existing therapies (hormone therapy, antibiotics, steroids) and putative novel therapies (prebiotics, postbiotics, vaginal microbiome transplant, vitamin supplementation, and live biotherapeutics) that can shift preterm birth-associated dysbiotic states to an optimal *Lactobacillus*-dominant and health-associated state. Antibiotics can directly inhibit pathogenic or preterm birth-associated bacteria, while pre-, pro-, and postbiotics, likely with lactobacilli or lactobacilli derivatives, may indirectly modify the cervicovaginal environment, making it inhospitable to pathogenic microbes. Vitamins and hormones can promote glycogen deposition and increase cell barrier stability, thereby promoting an overall homeostatic environment. These approaches require further investigation, specifically with regard to reproductive health outcomes.

Monitoring and potentially modulating the vaginal microbiome and cervicovaginal microenvironment before or early in pregnancy (150) can lead to better pregnancy outcomes (Fig. 3). Some potential therapy candidates have been used to ameliorate inflammatory states, such as steroids and hormonal therapy (151). Since a link between BV and PTB has been established, it is reasonable that treatments previously used for BV treatment can act as prophylactic therapies for PTB. Current methods for diagnosing and treating microbiome-related conditions are still evolving and have produced variable results, often based on studies with small participant cohorts (152). More recently, a paradigm shift in BV treatment now supports treating both partners to achieve sustained clinical resolution (122, 153). To determine the effectiveness and safety of these methods, larger and well-designed studies with geographically diverse and inclusive populations are fundamental. For this reason, the U.S. Preventive Services Task Force currently recommends against screening for BV to reduce the risk of PTB (154). The usage of vaginal microbiome transplantation (VMT) has been proposed (155) for preventing PTB. Preliminary data points to the beneficial and long-term impact of VMT in a recurrent miscarriage patient who had a healthy pregnancy and delivered the baby at term (152). That said, this is a single example, and more clinical trials are needed to assess efficacy and monitor long-term impacts on maternal-infant dyads. Other potential treatments involving live biotherapeutics have been proposed, including Lactin-V, which is being assessed for safety and tolerability in pregnant patients (156, 157). Further research aims to evaluate the role of biotherapeutics in preventing PTB (157), with potential implications for other adverse pregnancy outcomes.

Diet and vitamin supplementation have also been considered as a natural way to modulate the microbiome and could be a more accessible way to improve maternal-infant health in pregnancy (158). Prebiotics, probiotics (150), and postbiotics (128) have been used as therapy for inflammatory gastrointestinal diseases with promising or mixed results. These approaches, in addition to live biotherapeutics, should be considered possible candidates for future clinical research to assess long-term effects on the cervicovaginal and uterine environments (Box 1) as currently, there seems to be no differences observed when taking probiotics during pregnancy (159). Additionally, diagnostic developments (160), ranging from panels of novel microbial/metabolic biomarkers (161), microfluidic diagnostics (162), and monitoring genital inflammation, remain active areas of investigation and hold promise for improving accessibility and control in maternal and neonatal health (163).

Box 1. **Definitions of microbial modulators for improved health outcomes**

**Prebiotics** are microbial metabolites and byproducts of fermented dietary nutrients known to improve the host microbiota’s health. Prebiotics, when used as therapy, must meet specific criteria: they should resist digestion by gastric acid and enzymes, not be absorbed in the gut, be fermented by gut microbes, and selectively promote healthy gut bacteria. However, this definition may be soon updated as research investigates prebiotic development for the vagina, both alone or in conjunction with probiotic delivery.

**Probiotics** refer to living organisms that are utilized to support or maintain general health. Most probiotics are found in food products like yogurt but are also available for oral or intravaginal usage on their own. Probiotics are characterized by the following: must be alive and in large quantities, genetically identified, categorized according to current nomenclature, and show evidence of safety for general use plus some evidence of benefit with typically lower regulatory burden.

**Live Biotherapeutic Product (LBP)** refers to live organisms, such as bacteria designated to the prevention, treatment, or cure of a disease or condition of human beings and is not a vaccine. LBPs are subject to strict regulatory requirements. Several LBPs currently in clinical trials for gynecologic and obstetrics health include *Lactobacillus crispatus* CTV-05, *Lactobacillus rhamnosus* strain GG, and *Bifidobacterium lactis*.

**Postbiotics** are substances derived from microorganisms, including bacterial cell components, metabolites, proteins, or peptides. Postbiotics are often from the microorganism in which they are produced or purified and must be from known single or multiple organisms with genetic information and validated experimentally. Current postbiotics are heat-inactivated *Bifidobacterium bifidum* MIMBb75 and *Lactobacillus gasseri* CP2305, which have been shown to alleviate symptoms of irritable bowel syndrome and can provide relief for many women who report gastrointestinal symptoms during pregnancy.

As the field rapidly evolves and new strategies to modulate the microbiota emerge, it is essential to define the quality, purity, potency, safety, timing, and accessibility of these therapies, as well as who stands to benefit most and what adverse events may occur. Safety is particularly critical when considering the long-term effects of live biotherapeutics on host health, their impact on the microbiota, and the potential for horizontal transfer of antibiotic-resistance genes. While several microbiome-modulating interventions are available for healthy individuals, more evidence-based recommendations are still needed for their use in vulnerable populations, such as pregnant women and preterm newborns, to properly define risk-benefit profiles (164).

## CLINICAL ASPECTS AND IMPLICATIONS OF THE URINARY MICROBIOME IN PREGNANCY

In the urinary tract, decreased ureteral tone combined with mechanical compression of the ureters by the gravid uterus increases bladder residual volume, leading to urinary stasis and incontinence. These changes increase the risk of bacterial colonization and the development of urinary tract infections (UTIs) (165, 166). Typically, UTIs in pregnancy have a starting point on asymptomatic bacteriuria (ASB), with about 25% of ASB cases developing into upper UTI (pyelonephritis) (167).

Bacteriuria occurs in pregnant and nonpregnant women at similar rates, with a prevalence of 2–7% (168), and is often transient, particularly among women of reproductive age (169). The underlying reasons for the absence of symptoms in ASB are not well understood, although urinary immune host response is often detected (pyuria) (168). Thus, bacteriuria does not necessarily indicate a UTI. In addition, several factors contribute to UTIs in pregnancy, such as genitourinary abnormalities, primiparity, previous UTIs, diabetes, and low socioeconomic status (reviewed in 170).

Screening for ASB is a routine pregnancy test, typically performed once in the first or second trimester, with antibiotic treatment recommended if results are positive and the patient is considered at high risk for upper urinary infections (the current U.S. standard of care) (171, 172). However, relatively weak evidence supports ASB treatment for reducing adverse pregnancy outcomes related to upper UTI, such as low birth weight and PTB (165, 173–175). While ASB may still carry a risk of progression to upper UTI, overtreatment raises concerns about unnecessary risk of antimicrobial resistance. In addition, emerging evidence from mouse studies suggests that gut-associated dysbiosis due to antibiotic use during pregnancy can negatively affect offspring immune development such as antiviral immunity (69). Maternal gut dysbiosis has also been linked to altered placental metabolism, function, and immunity (14, 20, 63). Given these considerations, it has become increasingly important to understand the maternal urinary microbiome during pregnancy. ASB represents a clinically relevant and accessible condition that may serve as a proxy for exploring urobiome dynamics during pregnancy.

In non-pregnant women, the urobiome shows high inter-individual variability and is often asymptomatic (176). Advances in both culture-dependent and -independent methods have revealed greater microbial diversity than previously appreciated, with dominant genera including *Lactobacillus*, *Limosilactobacillus*, *Latilactobacillus*, and *Corynebacterium* (177, 178). The urobiome contains a range of microbes with diverse ecological roles, including pathogens, pathobionts, symbionts, commensals, and colonizers, reflecting a highly dynamic microbial environment (179, 180).

Importantly, the vaginal microbiota does not accurately represent the microbial composition of the lower urinary tract (urethra and bladder) (181), although some similarities exist between these sites (180). Moreover, the microbial communities of the female bladder mucosa differ from those found in urine samples (182). These distinctions underscore the importance of careful sampling strategies in urobiome studies (i.e., midstream clean catch urine sample *vs*. catheterization) (183). Shedding of bladder epithelial cells may also influence urinary microbial profiles, adding another layer of complexity to their interpretation (184). Finally, while 16S rRNA sequencing is highly sensitive, some organisms remain identifiable only by culture-based approaches (185). Accounting for relevant covariates that shape the urinary microbiome has been shown to improve the robustness of multivariable models when predicting clinical associations (186).

Throughout gestation, the composition of the maternal urobiome shifts in response to hormonal and immunological changes. The urobiome of first-trimester pregnant women is enriched for *Lactobacillus*, followed by *Gardnerella* (181), similar to non-pregnant women, who also harbor *Streptococcus*, *Staphylococcus*, and *Prevotella* (182, 187). In the second and third trimesters, *Lactobacillus* and *Prevotella* dominate the urobiome (188, 189). After delivery, the abrupt decline in estrogen and progesterone levels, along with postpartum inflammation, increases *Proteobacteria* abundance in the urobiome (189, 190). Urinary estrogen levels positively correlate with *Lactobacillus* and *Bifidobacterium* abundance (191). Urobiome alterations observed in pregnant women who conceived through IVF further support these patterns, with increased *Staphylococcus* and decreased *Lactobacillus*, which is typically enriched before pregnancy and associated with better IVF outcomes (47, 192).

Given that UTIs increase the risk of PTB, researchers have evaluated the predictive value of the second-trimester urobiome. This supports the concept of bacterial swapping or sharing among body sites (193, 194), exemplified by the *E. coli* strains that traffic between the gut, vagina, and bladder (e.g., the gut-bladder axis), establishing reservoirs for recurrent UTIs (195–197). While the urobiome composition during pregnancy does not predict PTB, specific bacteria, such as *Prevotella*, *Sutterella*, *Lactobacillus iners*, *Blautia*, *Kocuria*, *Lachnospiraceae*, and *Serratia marcescens*, have been associated with PTB risk. In healthy adolescent pregnancies (<17 years), *L. iners* and *L. kitasatonis* gradually increase with gestation age (198). In contrast, *L. iners* dynamics differ in adolescents with UTIs, rising from the first to the second trimester and declining thereafter, with recurrent infections showing a steady decrease across pregnancy (198). It remains unclear whether the presence of these low-abundance pathobionts or opportunistic pathogens is transitory or indicates an early shift over predominant health-associated urobiome. Similarities in urobiome composition between individuals with ASB and those with symptomatic infections suggest that the onset of urinary symptoms may be driven more by host immune responsiveness than by the mere presence of bacteria (176). In some cases, microorganisms may access atypical body sites and disturb existing biomes, thereby triggering disease (199). This phenomenon, known as covert pathogenesis, describes a situation in which the microbe responsible for initiating disease is no longer present at the time or location of disease manifestation (200). For example, the transient presence of *Gardnerella vaginalis* in the bladder has been shown to activate dormant *E. coli* infection in a mouse model (199). These findings suggest that the bladder microbiota is uniquely dynamic, with a heightened propensity for rapid shifts in microbial composition affecting its function. However, much remains to be understood about how pregnancy may influence a healthy urinary microbiome.

Local bladder immunity relies on draining lymph nodes to maintain tolerance to commensals, limit inflammation, and promote the clearance of infections (201). In a pregnancy model of UTI, bladder infection alone was shown to be sufficient to trigger PTB, uteroplacental inflammation (202), and intrauterine growth restriction (203). Remarkably, *E. coli* was found in the ileal lymph node of pregnant dams, a site that drains both the bladder and uterus. While direct lymphatic dissemination of *E. coli* from the bladder to the uteroplacental compartment remains unclear, this finding suggests a potential route for immune cross-talk that may explain how infection in the bladder could influence distal reproductive tissues.

Consistent with these findings, increased Th2 and Th17 cell populations were observed in the placenta of infected dams, even in the absence of bacterial colonization (202). This observation is particularly relevant given that *E. coli* can shift local T-cell responses from a Th1 to Th2 profile (204), a shift that may have broader implications for urobiome composition and immune regulation. Supporting this idea, women with recurrent UTIs often show loss of diversity of beneficial gut commensals, including species that produce anti-inflammatory SCFAs (196). These findings highlight a broader, yet poorly understood, role for the urobiome in influencing pregnancy outcomes.

## MATERNAL MICROBIOTA-PLACENTAL INTERACTIONS

The challenges in detecting the occasional presence of live microbes and distinguishing genuine microbial signals from contaminating noise in low biomass fetal samples have hindered further investigation of the fetal compartment (Box 2). These challenges also extend to other anatomical sites traditionally considered sterile, such as the blood (205), cerebrospinal fluid (206), liver (207, 208), and brain (209). Notably, the existence of a microbiota in defined sterile body sites, if true, would require a systematic review of biological concepts as discussed elsewhere (e.g., immunology, clinical microbiology and diagnostics, gnotobiology and developmental biology) (27).

Box 2. **Contrasting views on prenatal microbial colonization**

**The sterile womb hypothesis**

The sterility of the womb has been a prevailing belief, supported by the absence of detectable bacteria in amniotic fluid, cord blood, and placental tissues under standard sterile conditions. Advocates of this view maintain that such an environment is critical for preventing infections and ensuring the safe development of the fetus, whose immune system is not yet fully mature. Also, this view is supported by several studies reporting that bacterial signals come from sample contamination, “kitome,” and “splashome.” Moreover, fetal environment sterility is the basis for the derivation of gnotobiotic animal models. The sterility hypothesis underscores the importance of the birth process as the moment of initial microbial exposure, which is essential for the development of the neonatal immune system.

**The** ***in utero*** **colonization hypothesis**

On the other hand, the *in utero* colonization hypothesis has emerged from findings of microbial DNA within the placenta and amniotic fluid, challenging the notion of a sterile intrauterine environment. This hypothesis suggests that microbes or microbial products can access the womb, potentially influencing immune system maturation and priming the neonatal microbiome. This line of thought is supported by evidence suggesting that the healthy placenta (basal plate) can harbor bacteria without overt inflammation and the presence of BEVs, both hinting at possible maternal-fetal microbial transfer mechanisms. Importantly, these observations may also be explained by the translocation of maternal bacterial DNA across the maternal-fetal interface, rather than the establishment of fetal microbiome.

New studies demonstrating that bacterial extracellular vesicles (BEVs) can carry microbial DNA might explain the finding of microbial signals, but they also complicate nucleic acid-based approaches commonly used in microbiome studies, including those of the placenta (210).

While the existence of a fetal microbiota remains unresolved, earlier human studies suggest that microbial components, irrespective of their origin, may modulate fetal development *in utero* and prime early immune responses (24, 25, 29, 30, 211, 212). For example, *Micrococcus luteus*, found enriched in the human fetal intestine, was shown to modulate mucosal immunity by inducing tolerogenic antigen-presenting cells (APCs), IL-10 secretion, and inhibiting IFN-γ production by fetal intestinal memory T cells *in vitro* (24, 213). *Staphylococci* and *Lactobacilli* isolates from the human fetus intestine were shown to prime fetal APCs isolated from the mesenteric lymph node and induce fetal T-cell proliferation, expansion, and cytokine secretion (TNF and IFN-γ) (25). Other bacterial genera also found in the fetal gut are *Streptococcus*, *Enterococcus*, *Bacteroides, Bifidobacterium*, *Prevotella*, and *Finegoldia* (24, 25). The potential of these bacteria to initiate the development of fetal mucosal immunity is not yet clear. Notably, the presence of memory-like CD4^+^ T cells in the fetal intestine is consistent with the exposure to foreign antigens *in utero* (214, 215). Alternatively, transplacental transfer of maternal immunoglobulins and microbiota-derived metabolites can also induce fetal immune responses (27). It is noteworthy that gut bacterial extracellular vesicles coated with IgA have been shown to induce ulcerative colitis in non-pregnant individuals and in a mouse model (216), suggesting that similar Ig-coated vesicles could cross the placenta alongside maternal antibodies. Together, these studies highlight a role of maternal and microbial factors in shaping fetal development, possibly separate from direct colonization.

Although the mechanisms underlying the transplacental transfer of metabolites are not fully understood, murine maternal IgG antibodies are known to facilitate the transfer of microbial compounds to the developing fetus, including ligands for the aryl hydrocarbon receptor (AhR) that was shown to induce the differentiation of NKp46+ innate lymphoid cells in the fetal intestine (211). The translocation of maternal microbiota components to the fetus can also occur through bacterial-derived vesicles, as recently detected in the amniotic fluid, placenta, and first-pass meconium (76, 210, 217). These findings in humans are important because bacterial extracellular vesicles are more likely to cross the placenta than whole-cell bacteria, potentially explaining discordant results observed in 16S rRNA gene sequencing between fetal samples and culture isolates (24). In addition, it highlights a putative mechanism underlying the long-standing association between bacterial infections (e.g., urinary tract infections, periodontitis) and PTB risk (218, 219).

There is evidence that colonization may occur during the first trimester, as recent studies have reported the presence of bacteria in human placental and fetal tissues as early as 10 weeks of gestation (22, 25, 26, 220–222). Notably, although the placental tissues of fetal origin (i.e., chorionic villi and plate, amnion, and chorion) have been extensively studied, research on placental tissues of maternal origin (i.e., the basal/decidual plate and decidua) is less frequent. The difficulty of studying placental tissues of maternal origin is attributed to their structure and direct contact with the maternal blood, which bathes the placenta (intervillous space) and can introduce a source of bacterial particles (dead or alive) or remnants of a low-grade bacterial infection (223). Therefore, whether the maternal-fetal interface has a microbiota remains poorly understood and requires further investigation.

In contrast, it is well known that pathogenic bacteria can colonize the placenta, leading to adverse pregnancy outcomes (224). However, it has been shown that both term and preterm human placentas, specifically the basal plate, can host bacteria and biofilm-like clusters of bacteria without showing signs of overt infection as detected by multiple stains (225–227). The fact that bacteria are not always present in the placenta basal plate and are not always linked to disease often leads to missed detection in histopathological examinations. Whether the bacteria in the basal plate are commensals or persist in a viable but non-cultivatable state is unknown (228). Also, the factors that inhibit bacterial progression from becoming a clinical infection are yet to be fully understood. *Ex vivo* experiments on human basal plate explants, specifically bacterial infection with *E. coli* and *Listeria monocytogenes*, suggested particular affinity (tropism) for HLA-G+ extravillous trophoblast cells (226). Additionally, it has been reported that *Ralstonia insidiosa*, found in the basal plate of healthy term placentas, can induce HLA-G secretion and modulate decidual natural killer cells (227, 229). This field continues to evolve slowly as new studies emerge to establish residence of specific bacteria and define their symbiotic relationships. Further investigation into the spatial distribution of these bacteria can yield insights that are critical not only to understanding their presence but also for elucidating their biological roles in pregnancy.

## CONCLUSIONS AND PERSPECTIVES

Human pregnancy is a fascinating and complex phenomenon in which the maternal microbiome and traditional factors (e.g., genetics, immunology, physiology, and environment) contribute to its development. Changes in the gut and cervicovaginal microbial communities have evidenced the maternal microbiome’s impact on pregnancy outcomes (61, 230). Whether pathological changes impact long-term maternal and neonatal health is still unknown. Other bacterial communities, such as the oral, nasal, and skin, have been shown to influence newborn health, but their role during pregnancy is yet to be defined (231–233).

The non-pathological and pathological microbiota have been traditionally assessed by microbial diversity (loss or gain) and representation of bacterial genera/species (dominance or depletion) in healthy and pathological pregnancies. It is important to note that the symbiotic relationship between microbes and the developing fetus has been tested only with a limited number of culturable bacteria (24, 25, 227), implying that our understanding is incomplete, particularly of less commonly studied bacterial species and commensal gut mycobiota (234). Also, understanding of how individual bacteria influence others at a community level is far from being understood. Because the vast majority of microbes are not culturable (235) and 16S rRNA gene sequencing does not allow the assessment of bacterial viability, there is a growing need to develop more sophisticated and integrative techniques such as cultivation and computer modeling (236, 237).

The lack of mechanistic understanding of how microbiota affects pregnancy, individually and collectively, and what influences their population dynamics and structure remains a significant challenge in the field. In the context of pregnancy, organ-on-chip models (vagina-on-chip) and organoids for the study of intestinal and vaginal microbiota-host interactions will provide important clues to their impact on pregnancy and potential therapeutics (238, 239). From a personalized medicine viewpoint, the intra-individual and temporal microbiome differences require revisiting the concept of healthy microbiota (240), as inherent biological, social determinant, and cultural differences among human populations affect the microbiome composition (241). Although calls for more diverse participant cohorts have increased, significant gaps remain in the geographic representation of microbiome research relevant to obstetric health. Most existing studies originate from North America, Europe, and Northeast Asia, limiting the ability to generalize findings globally. Equitable and collaborative partnerships with low- and middle-income countries, where unmet maternal health needs are often greatest, are essential for expanding geographic diversity and improving our understanding of environmental and geographic impact on the microbiome during pregnancy (242, 243). Investigating these aspects is particularly important for future research, especially in understanding reproductive health and the risk of adverse pregnancy outcomes (244, 245). While targeted therapies aiming to modulate specific microbiota (156), clear infections (246), or genetically engineer others are emerging (247), the long-term effects on maternal and neonatal health are still unknown. Adding to these scientific challenges are the societal, legal, and ethical considerations, such as ensuring equitable access to new treatments across different communities, establishing clear regulatory frameworks for the safe application of microbiome interventions, and navigating the ethical complexities of informed consent and the potential impact on the fetus and newborn health (248).

While the ongoing debate over the *in utero* colonization hypothesis raised several fundamental questions (27, 249, 250), a more diverse and non-biased consensus from experts supporting the *in utero* colonization is still needed to move the field forward (251). Additionally, we advocate for the integration of multi-omics approaches (252), including (but not limited to) metagenomics, metabolomics, and transcriptomics, to determine how host-microbe and inter-site microbial interactions engage in dynamic crosstalk and trafficking during pregnancy, with certain niches potentially serving as reservoirs that influence maternal-fetal health outcomes. A practical starting point would be to analyze temporal changes in microbiota composition across multiple body sites during pregnancy, along with pregnancy metabolite profiles and/or bacterial-derived exosomes, to better define host-microbe and inter-site microbial interactions. Finally, an intentional and collaborative effort to establish universal standards and practices for maternal microbiome studies could help generate more accurate, reproducible, and comprehensive data (253), ultimately enriching our understanding of the microbiome’s role in maternal-fetal health. Collectively, this knowledge could lead to novel approaches for enhancing maternal-fetal health, improving disease management, and developing novel therapeutic strategies.
